# Circular RNA USP1 regulates the permeability of blood‐tumour barrier via miR‐194‐5p/FLI1 axis

**DOI:** 10.1111/jcmm.14735

**Published:** 2019-10-26

**Authors:** Yang Gao, Peiqi Wu, Yawen Ma, Yixue Xue, Yunhui Liu, Jian Zheng, Xiaobai Liu, Qianru He, Jun Ma, Libo Liu, Ping Wang

**Affiliations:** ^1^ Department of Neurobiology College of Life Science China Medical University Shenyang China; ^2^ Key Laboratory of Cell Biology Ministry of Public Health of China China Medical University Shenyang China; ^3^ Key Laboratory of Medical Cell Biology Ministry of Education of China China Medical University Shenyang China; ^4^ Laboratory of Digital Health Medaxis Technology Co., Ltd. Chengdu China; ^5^ Department of Neurosurgery Shengjing Hospital of China Medical University Shenyang China; ^6^ Liaoning Clinical Medical Research Center in Nervous System Disease Shenyang China

**Keywords:** blood‐tumour barrier, circ‐USP1, FLI1, miR‐194‐5p, permeability

## Abstract

Recent studies indicate circular RNAs are related to dysregulation of vascular endothelial cell function, yet the underlying mechanisms have remained elusive. Here, we characterized the functional role of circular RNA USP1 (circ‐USP1) in the regulation of the blood‐tumour barrier (BTB) permeability and the potential mechanisms. In the current study, the circ‐USP1 expressing level was up‐regulated in glioma cerebral microvascular endothelial cells (GECs) of the BTB model in vitro. Knockdown of circ‐USP1 disrupted the barrier integrity, increased its permeability as well as reduced tight junction‐related protein claudin‐5, occludin and ZO‐1 expressions in GECs. Bioinformatic prediction and luciferase assay indicated that circ‐USP1 bound to miR‐194‐5p and suppressed its activity. MiR‐194‐5p contributed to circ‐USP1 knockdown‐induced increase of BTB permeability via targeting and down‐regulating transcription factor FLI1. Furthermore, FLI1 regulated the expressions of claudin‐5, occludin and ZO‐1 in GECs through binding to their promoter regions. Single or combined treatment of circ‐USP1 and miR‐194‐5p effectively promoted anti‐tumour drug doxorubicin across BTB to induce apoptosis of glioma cells. Overall, this present study identified the crucial regulation of circ‐USP1 on BTB permeability via miR‐194‐5p/FLI1 axis‐mediated regulation of tight junction proteins, which might facilitate the development of therapeutics against human gliomas.

## 1 INTRODUCTION

Glioma originated from glial cells or precursor cells, consists of 30% of all brain tumours and 80% of malignant primary brain tumours.^1^ At present, the main treatment methods for glioma are surgery combined with radiotherapy and chemotherapy. However, due to the existence of the blood‐tumour barrier (BTB), large molecules of chemotherapy drugs cannot enter the tumour tissue, which dramatically influences chemotherapy delivery.^2^ Therefore, selectively opening of BTB, improving the permeability of BTB and increasing anti‐tumour medicine concentration in the brain tumour tissue provide a new approach to the treatment of glioma.

Circular RNAs (circRNAs) from back‐spliced exons or intron‐derived RNA have been recognized as a relatively new family of non‐coding RNAs. They are found mainly in the cytoplasm and can be sorted into exosomes.^3^ There is accumulating evidence indicates circRNAs serve as microRNA sponges or regulators of gene splicing and transcription to regulate gene expression.^4^, ^5^ However, the functions of thousands of described circRNAs remain unclear. Recently, abnormal circRNA expressions have been demonstrated in various cardiovascular diseases and cancers, which are related to vascular dysfunction.^6^, ^7^, ^8^ Knockdown of cZNF609, which is highly enriched in endothelial cells, diminished retinal vessel loss and inhibited pathological angiogenesis in vivo.^9^ CircRNA USP1 (circ‐USP1, also known as hsa_circ_0000080 according to circBase), which is located at chr1p31, is derived from the back‐splicing of exon 6‐8 of ubiquitin‐specific peptidase 1 (USP1) gene. Circ‐USP1 is significantly up‐regulated in our microarray screen detected by comparing human cerebral microvascular endothelial cell line (ECs) with endothelia cells co‐cultured with glioma (GECs). Moreover, USP1 is highly expressed in glioblastoma, particularly in enriched glioblastoma stem‐initiating cells.^10^ Hence, circ‐USP1 might be related to the dysregulation of endothelial cell functions.

MicroRNAs (miRNAs) are a kind of small conserved non‐encoding RNA molecules that are involved in the post‐transcriptional gene regulatory mechanism. MiRNAs have also been known to participate in a variety of physiological and pathological processes.^11^, ^12^ Moreover, accumulating data indicate that miRNAs play crucial roles in regulating endothelial barrier function.^13^, ^14^, ^15^ Decreased expression of miR‐194‐5p has been reported in various types of malignancy, including glioma, hepatoma carcinoma, gallbladder carcinoma and acute myeloid leukaemia.^16^, ^17^, ^18^, ^19^ Overexpressed miR‐194‐5p in non–small‐cell lung cancer suppresses cell migration, invasion and metastasis.^20^ These reports indicate that miR‐194‐5p functions as a tumour suppressor in various human malignancies. MiR‐194‐5p could also modulate astrocyte‐endothelial cell transition by driving expression of endothelial‐specific genes, which indicated that miR‐194‐5p might play a role in regulating endothelial cell function.^21^ MiR‐194‐5p was predicted to potentially target the circ‐USP1 using CircInteractome database (https://circinteractome.nia.nih.gov/). Nevertheless, whether miR‐194‐5p might be involved in regulating BTB permeability remains to be investigated.

The highly conserved ETS transcription factors share a winged helix‐turn‐helix DNA‐binding domain.^22^ Friend leukaemia virus integration 1 (FLI1), as one of the ETS members, was initially uncovered as an oncogene as it is involved in retrovirus‐induced haematological tumours in mice.^23^ In human tissues, FLI1 is abnormally expressed in some solid tumours, including Ewing sarcoma, breast cancer and astrocytoma.^24^, ^25^, ^26^ In endothelial cells, FLI1 is a regulator of vessel maturation and stabilization via modulating expressions of genes involved in maintaining vascular homoeostasis and integrity.^27^, ^28^ However, a little attention has been directed to clarify the possible role of FLI1 in GECs and BTB permeability.

In our study, we explored the expressions of circ‐USP1, miR‐194‐5p as well as FLI1 in GECs and elucidated their roles in the regulation of barrier permeability. We revealed that silencing circ‐USP1 could increase the BTB permeability via miR‐194‐5p/FLI1‐mediated regulation, which would provide a new therapeutic strategy of glioma.

## 2 MATERIALS AND METHODS

### 2.1 Cell lines and culture

The immortalized human cerebral microvascular endothelial cell line (hCMEC/D3, ECs) was kindly supplied by Dr Couraud (Institut Cochin, Paris, France). ECs were cultured as described previously.^29^ Human glioma cell line U87MG and human embryonic kidney 293T (HEK293T) cell line were purchased from the Shanghai Institutes for Biological Sciences Cell Resource Center and were cultured in Dulbecco's modified Eagle medium of high glucose containing 10% foetal bovine serum, 100 U/mL penicillin and 100 μg/mL streptomycin (Life Technologies).

### 2.2 Establishment of BTB model in vitro

The in vitro BTB model was established by co‐culture of ECs and U87 cells as described previously.^29^, ^30^, ^31^ Briefly, the U87 cells at a density of 2 × 10^4^ per well were seeded in the six‐well plate. Two days later, the ECs at a density of 2 × 10^5^ per well were seeded onto the upper side of the Transwell insert (0.4 μm pore size; Corning) coated freshly with 150 μg/mL of Cultrex Rat Collagen I (R&D Systems). The inserts were placed in the well of the six‐well plates containing U87 glioma cells and co‐cultured for 4 days with prepared endothelial basal medium 2, and the medium was changed every 2 days. After co‐culture with U87 glioma cells for 4 days, the ECs were called GECs.

### 2.3 Circular RNA microarray analysis

Total RNA was extracted with Trizol reagent (Life Technologies). Circular RNA microarray analysis was performed by Kanchen Corporation. Microarray hybridization was performed according to the Arraystar's standard protocols.

### 2.4 Real‐time PCR assay

The expression of circ‐USP1 was detected using One Step PrimeScript™ RT‐PCR Kits (Takara, RR064A). The probe and primers of circ‐USP1 and GAPDH were synthesized from Takara. The expression of linear USP1 was assessed by SYBR Premix Ex Taq and TaqMan gene expression assay kit (Applied Biosystems). The miR‐194‐5p and U6 expressions were determined using TaqMan MicroRNA Reverse Transcription kit and Taqman Universal Master Mix II (Applied Biosystems). Relative expression values were normalized and calculated with the relative quantification (2^−ΔΔCt^) method. Probes and primers used for quantitative PCR (qPCR) were listed in Table S1.

### 2.5 Cell transfection

The short‐hairpin circ‐USP1 (circ‐USP1 (−)), linear USP1 (lin‐USP1 (−)) and FLI1 (FLI1 (−)) plasmids, and their respective negative control (NC), the non‐targeting sequence, were ligated into a pGPU6/GFP/Neo plasmid (GenePharma). FLI1 full length (FLI1 (+)) vector and its respective NC were reconstructed into a pIRES2‐EGFP plasmid (GenScript). AgomiR‐194‐5p (miR‐194‐5p (+)), antagomiR‐194‐5p (miR‐194‐5p (−)) and their corresponding NC were synthesized (GenePharma).

ECs were transfected with Lipofectamine LTX and Plus reagent (Life Technologies). G418 was used to select the stably transfected cells. The sequences for shRNA targeting circ‐USP1, FLI1 and NC were showed in Table S2.

### 2.6 Transendothelial electric resistance (TEER) assays and horseradish peroxidase (HRP) assays

Before TEER assay was conducted using Millicell‐ERS apparatus (Millipore), the inserts with ECs and U87 cells co‐culture were leave in room temperature for 30 minutes. The medium was refreshed before the measurement. After subtracting the background resistance, the final TEER value (Ω·cm^2^) was calculated by multiplying the remained barrier resistance with the surface area of the Transwell insert.

After the BTB model was constructed, HRP (10 μg/mL, Sigma‐Aldrich) was added into the upper chamber of the Transwell system. One hour later, 5 μL of culture medium was collected from the lower chamber. The final HRP flux was presented as pmol·cm^−2^·h^−1^.

### 2.7 Western blotting and immunofluorescence assays

Western blotting was performed as previously described.^29^ Primary antibodies against GAPDH (Proteintech), FLI1 (Abcam), ZO‐1 (Life Technologies), occludin (Abcam) and claudin‐5 (Life Technologies) were used. Immunoblots were visualized using an enhanced chemiluminescence kit (ECL; Santa Cruz Biotechnology) and detected by MicroChemi 4.2 Bio Imaging System (DNR Bio‐Imaging Systems). The integrated light density values (IDV) were calculated and normalized with those of GAPDH. Distributions of claudin‐5, occludin and ZO‐1 were examined using immunofluorescence as reported previously.^29^

### 2.8 Dual‐luciferase reporter assay

The fragments of circ‐USP1, lin‐USP1 and FLI1 3′‐UTR containing the potential miR‐194‐5p binding sites as well as their mutant binding sites were cloned into the pmirGlo Dual‐luciferase miRNA Target Expression Vector (Promega) to construct the reporter vector (Generay Biotech Co.). HEK‐293T cells were cotransfected with the above pmirGLO vectors and agomiR‐194‐5p or agomiR‐194‐5p‐NC using Lipofectamine 3000 Reagents (Life Technologies). The relative firefly luciferase activity was determined 48 hours after transfection, and firefly luciferase activity was normalized by renilla luciferase activity.

### 2.9 RNA immunoprecipitation (RIP) assay

RNA immunoprecipitation assays were conducted according to the instruction of the Magna RIP RNA‐Binding Protein Immunoprecipitation Kit (Millipore). Briefly, cell lysates were incubated with RIP immunoprecipitation buffer containing magnetic beads conjugated with human anti‐Ago2 antibody, and normal mouse IgG. Samples were incubated with Proteinase K, and then, immunoprecipitated RNA was purified and applied to qPCR.

### 2.10 Chromatin immunoprecipitation (ChIP) assay

Chromatin immunoprecipitation assay was conducted using Simple ChIP Enzymatic Chromatin IP Kit (Cell signaling Technology) as previously described.^29^ 2% aliquots of lysates were used as input control, and the remaining lysates were immunoprecipitated with normal IgG or FLI1 antibody. Immunoprecipitated DNA was amplified using PCR by the primers listed in Table S3.

### 2.11 Analysis of apoptosis by flow cytometry

After BTB models were established in vitro, 10 µM of DOX (Beyotime Institute of Biotechnology) or 0.1% DMSO (as the control group) was added to the upper chamber of Transwell. The apoptosis rates of U87 cells were detected 12 hours later. Annexin V‐PE/7‐AAD apoptosis detection kit (Southern Biotech) was used to detect cell apoptosis according to the manufacturer's instructions. The U87 cells in the lower chamber were resuspended with Annexin V‐bounding buffer after washed with phosphate‐buffered saline and centrifuging twice. Resuspended cells were stained with Annexin V‐PE/7‐AAD for 15 minutes in the dark at room temperature. Cell samples were obtained by FACScan (BD Biosciences) to obtain the apoptotic fractions and analysed by CELL Quest 3.0 software. Early apoptosis is defined by Annexin V+/PI− staining (lower right quadrant, LR), and late apoptosis is defined by Annexin V+/PI+ staining (upper right quadrant, UR).

### 2.12 Statistical analysis

Experimental data were presented as the mean ± standard deviation and performed the statistical analysis using Student's *t* test (two‐tailed) and one‐way analysis of variance (ANOVA) with GraphPad Prism 5 (GraphPad Software). Differences were considered to be statistically significant when *P* < .05.

## 3 RESULTS

### 3.1 Silence of circ‐USP increased BTB permeability via reducing tight junction‐related protein expressions in GECs

**Figure 1:**
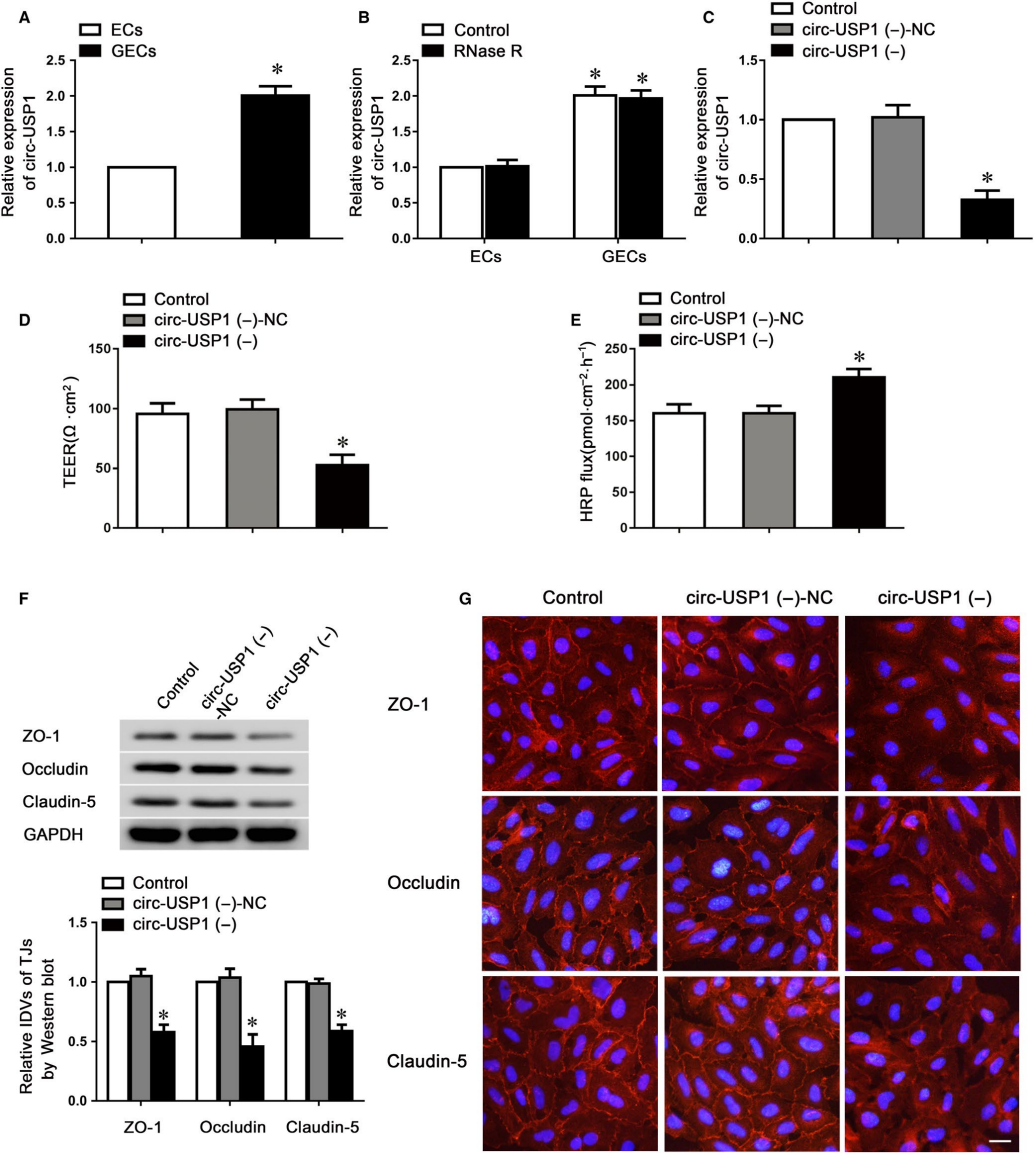
Circ‐USP1 regulated BTB permeability and the expression of tight junction‐related proteins in GECs. A, Relative circ‐USP1 expression in ECs and GECs by qRT‐PCR. B, Relative circ‐USP1 expression in ECs and GECs after RNase R treatment. Data represented as mean ± SD (n = 5). *P < .05 vs. ECs group. C, Relative expression of circ‐USP1 was evaluated using qRT‐PCR in the GECs with the circ‐USP1 knockdown. The BTB permeability and integrity was evaluated using TEER values (D) and HRP flux (E) in the BTB model with the circ‐USP1 knockdown. F, Western blot assay was conducted to detect the effect of circ‐USP1 knockdown on the expression of tight junction‐related proteins. Data represented as mean ± SD (n = 5). *P < .05 vs. circ‐USP1 (−)‐NC group. G, Immunofluorescent staining of tight junction‐related proteins in GECs with the circ‐USP1 knockdown. Scale bar represents 20 μm

To uncover the function of circular RNA in endothelial cell dysregulation, microarray analysis was conducted to investigate the expression of circular RNA in GECs after the BTB models were successfully constructed. The results showed circ‐USP1 was the most up‐regulated circular RNA in GECs (Figure S1). To further confirm this result, the endogenous expression of circ‐USP1 in GECs was validated using TaqMan qRT‐PCR. The expression of circ‐USP1 was highly enriched in GECs compared to ECs (Figure 1A). The expression of linear USP1 (lin‐USP1) was detected as well; no significant difference was found between ECs and GECs (Figure S2A). To further confirm the circular characteristics of circ‐USP1, the enzyme RNase R which does not act on circular RNA was used. As expected, circ‐USP1 was resistant to RNase R digestion, whilst lin‐USP1 was significantly degraded (Figure 1B and Figure S2B). Furthermore, shRNA directed against the back splice sequence of circ‐USP1 significantly decreased the expression of circ‐USP1 by 72% and did not affect the expression of lin‐USP1 (Figure 1C and Figure S2C). ShRNA targeted to the exonic sequences not shared by circ‐USP1, only markedly knocked down the lin‐USP1 expression (Figure S2D,E).

To further investigate whether circ‐USP1 could regulate barrier function, the stable circ‐USP1‐knockdown ECs were used to establish the BTB model in vitro. TEER and HRP flux assays were performed to assess the barrier integrity and permeability. Compared to the circ‐USP1 (−)‐NC group, the TEER value was markedly reduced and HRP flux was markedly increased in the circ‐USP1 (−) group (Figure 1D,E), indicating that circ‐USP1 knockdown disrupted barrier integrity and increased its permeability. To verify the underlying mechanisms of circ‐USP1 during this regulatory process, the expressions of claudin‐5, occludin and ZO‐1 were assessed using western blot. Data indicated that the circ‐USP1 (−) group presented significantly lower claudin‐5, occludin and ZO‐1 expressions compared with the circ‐USP1 (−)‐NC group (Figure 1F). Furthermore, immunofluorescence analysis confirmed that in circ‐USP1 (−)‐NC group, more continuous distributions of tight junction‐related proteins were observed. However, claudin‐5, occludin and ZO‐1 in circ‐USP1 (−) group distributed discontinuously on the boundaries of cells (Figure 1G). Taken together, these data demonstrated that knockdown of circ‐USP1 impaired the barrier integrity, increased permeability and reduced tight junction‐related protein expressions in GECs.

### 3.2 MiR‐194‐5p was involved in the regulation of BTB permeability and tight junction‐related protein expressions in GECs

**Figure 2:**
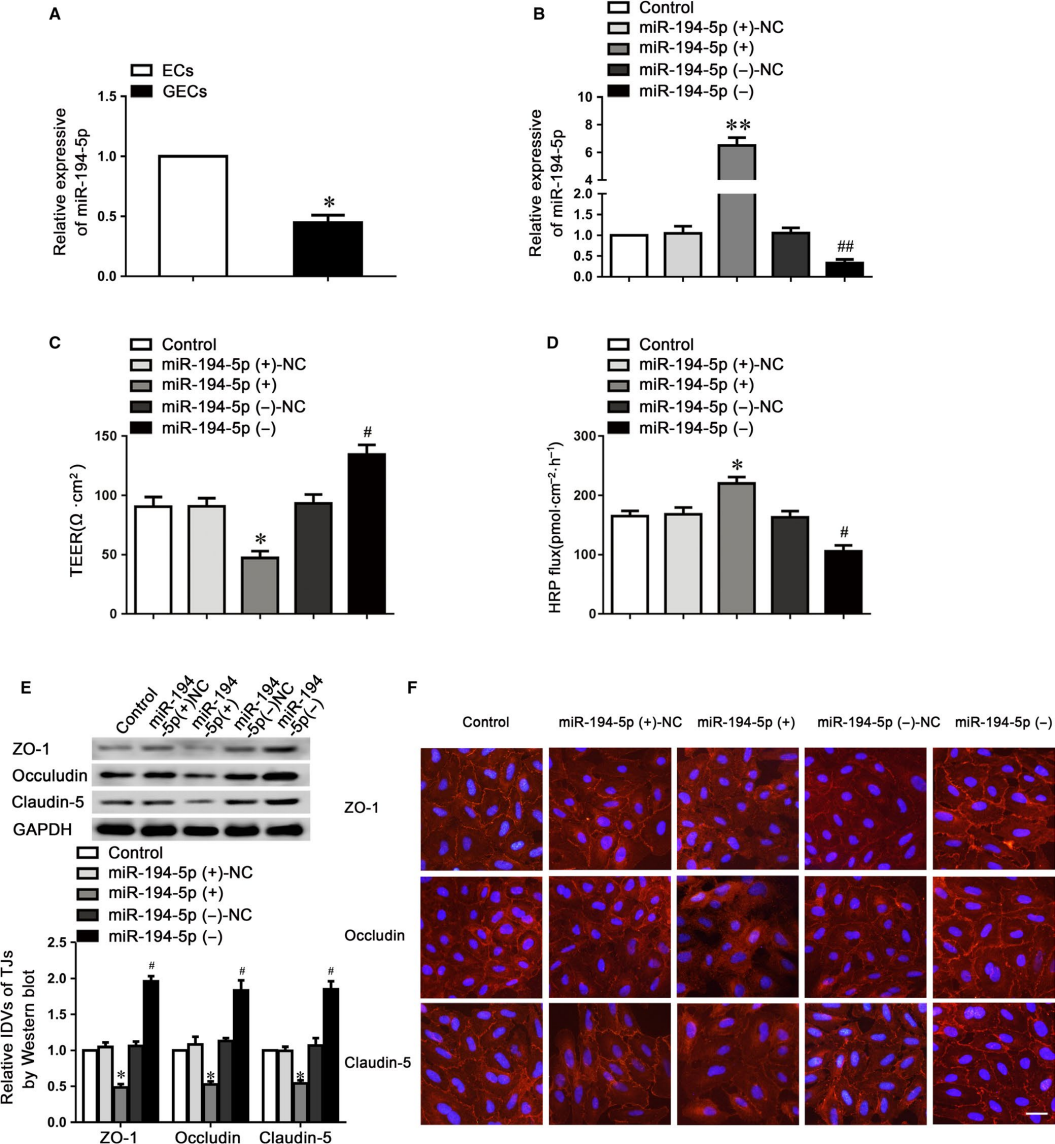
MiR‐194‐5p regulated BTB permeability and the expression of tight junction‐related proteins in GECs. A, Relative miR‐194‐5p expression in ECs and GECs by qRT‐PCR. Data represented as mean ± SD (n = 5). *P < .05 vs. ECs group. B, Relative expressions of miR‐194‐5p were evaluated using qRT‐PCR in the GECs with miR‐194‐5p overexpression or knockdown. Data represented as mean ± SD (n = 5). **P < .01 vs. miR‐194‐5p (+)‐NC group, ##*P* < .01 vs. miR‐194‐5p (−)‐NC group. Effects of miR‐194‐5p on TEER values (C) and HRP flux (D) in the BTB model in vitro. E, Effect of miR‐194‐5p on the expression of tight junction‐related proteins by western blot assay. Data represented as mean ± SD (n = 5). *P < .05 vs. miR‐194‐5p (+)‐NC group, ##*P* < .01 vs. miR‐194‐5p (−)‐NC group. F, Immunofluorescent staining of tight junction‐related proteins in GECs with miR‐194‐5p overexpression or knockdown. Scale bar represents 20 μm

To examine the mechanism underlying circ‐USP1 knockdown increases BTB permeability, we presumed that circ‐USP1 might act as a miRNA sponge to adjust gene expression. To clarify which miRNAs might bind to circ‐USP1, we assessed putative miRNA‐binding sites on the circ‐USP1 sequence using CircInteractome database. Among the 48 miRNAs candidates, miR‐194‐5p, which was recognized by circ‐USP1 with an 8mer seed type, showed the highest context score percentile among the candidates belonging to the broadly conserved and conserved miRNA families. In GECs, miR‐194‐5p expression was decreased compared with ECs (Figure 2A). Then, the effects of miR‐194‐5p overexpression and knockdown on the barrier integrity and permeability were assessed using TEER and HRP flux assays. The overexpression and knockdown levels of miR‐194‐5p were assessed by qRT‐PCR (Figure 2B). As indicated in Figure 2C,D, the miR‐194‐5p (+) group exhibited a markedly reduced TEER value, and an obviously up‐regulated HRP flux value, compared to the miR‐194‐5p (+)‐NC group. The opposite tendency of TEER and HRP flux values was observed in the miR‐194‐5p (−) group.

We subsequently investigated claudin‐5, occludin and ZO‐1 protein expression in GECs using Western blotting (Figure 2E). Results indicated that compared with the NC group, the tight junction‐related proteins expressions were markedly reduced in the miR‐194‐5p (+) group, whilst miR‐194‐5p knockdown significantly up‐regulated the expression of these proteins. Further, immunofluorescence analysis revealed the distribution of claudin‐5, occludin and ZO‐1 on the boundaries of ECs in the control group was closer to linear and was discontinuously localized at tight junctions in the miR‐194‐5p (+) group (Figure 2F). Therefore, the data above indicated that miR‐194‐5p impaired the integrity, increased barrier permeability via reducing claudin‐5, occludin and ZO‐1 expressions in GECs.

### 3.3 Circ‐USP1 bound to miR‐194‐5p to regulate BTB permeability

**Figure 3:**
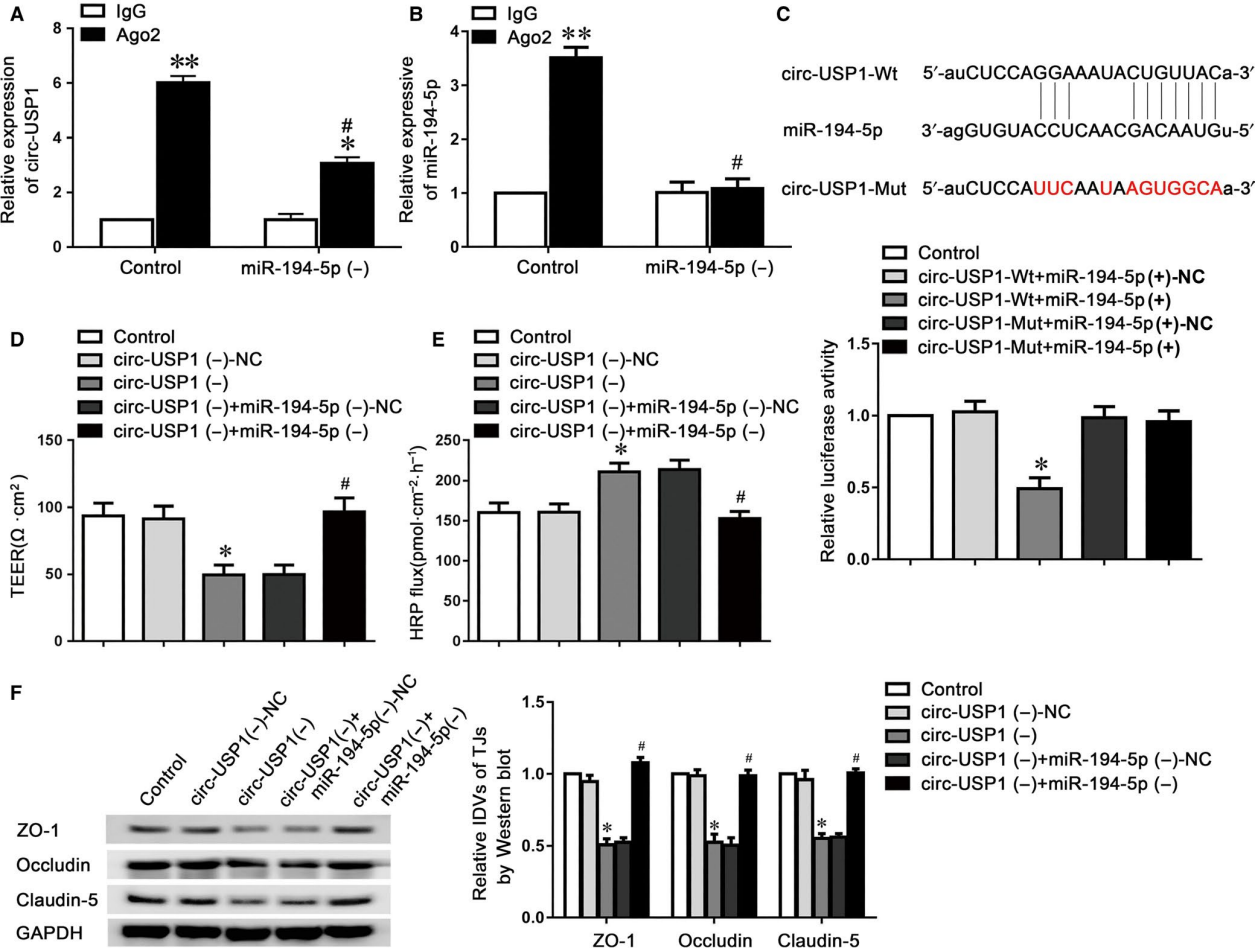
The interaction between circ‐USP1 and miR‐194‐5p was involved in the regulation of BTB permeability. RNA immunoprecipitation assay was performed with normal IgG or Ago2 antibody. Relative expression levels of circ‐USP1 (A) and miR‐194‐5p (B) were determined by qRT‐PCR. Data represented as mean ± SD (n = 5). *P < .05, **P < .01 vs. respective IgG group. #*P* < .05 vs. Ago2 in the control group. C, Relative luciferase activity was performed by dual‐luciferase reporter assay. Data represented as means ± SD (n = 5). *P < .05 vs. circ‐USP1‐Wt+miR‐194‐5p(+)‐NC group. Effects of circ‐USP1 and miR‐194‐5p knockdown on TEER values (D) and HRP flux (E) in the BTB model in vitro. F, Western blot assay to evaluate the effect of circ‐USP1 and miR‐194‐5p knockdown on the expressions of tight junction‐related proteins. Data represented as mean ± SD (n = 5). *P < .05 vs. circ‐USP1 (−)‐NC group, #*P* < .05 vs. circ‐USP1 (−)+miR‐194‐5p (−)‐NC group

MiRNAs were identified to regulate mammalian gene expression at post‐transcriptional levels by binding to Ago2. To investigate whether circ‐USP1 possesses miR‐194‐5p‐related functions by forming a complex with Ago2, RIP assay was performed. The data showed that circ‐USP1 and miR‐194‐5p were significantly enriched in Ago2 immunoprecipitate compared to that of IgG. The miR‐194‐5p knockdown markedly down‐regulated circ‐USP1 and miR‐194‐5p levels immunoprecipitated with Ago2 (Figure 3A,B). To verify the prediction that circ‐USP1 was targeted by miR‐194‐5p, the dual‐luciferase reporter assay was performed. The relative luciferase activity in the circ‐USP1‐Wt+miR‐194‐5p (+) group was reduced by 50%, compared to that in the circ‐USP1‐Wt+miR‐194‐5p (+)‐NC group. There is no difference between circ‐USP1‐Mut+miR‐194‐5p (+) and circ‐USP1‐Mut+miR‐194‐5p (+)‐NC group (Figure 3C). Moreover, no binding site was found between lin‐USP1 and miR‐194‐5p.

To further explore whether circ‐USP1 interact with miR‐194‐5p to regulate BTB permeability, antagomiR‐194‐5p was transfected into the stable circ‐USP1 knockdown GECs. Then, TEER and HRP flux assays were investigated. As indicated in Figure 3D,E, the reduced miR‐194‐5p markedly reversed the circ‐USP1 knockdown‐induced down‐regulation in TEER value and increase in HRP flux as well. Moreover, the expressions of claudin‐5, occludin and ZO‐1 were assessed using western blot assay, which revealed that the low expressing levels of claudin‐5, occludin and ZO‐1 induced via circ‐USP1 knockdown were markedly reversed in the circ‐USP1 (−)+miR‐194‐5p (−) group, compared to circ‐USP1 (−)+miR‐194‐5p (−)‐NC group (Figure 3F).

### 3.4 FLI1 regulated BTB permeability via tight junction‐related proteins

**Figure 4:**
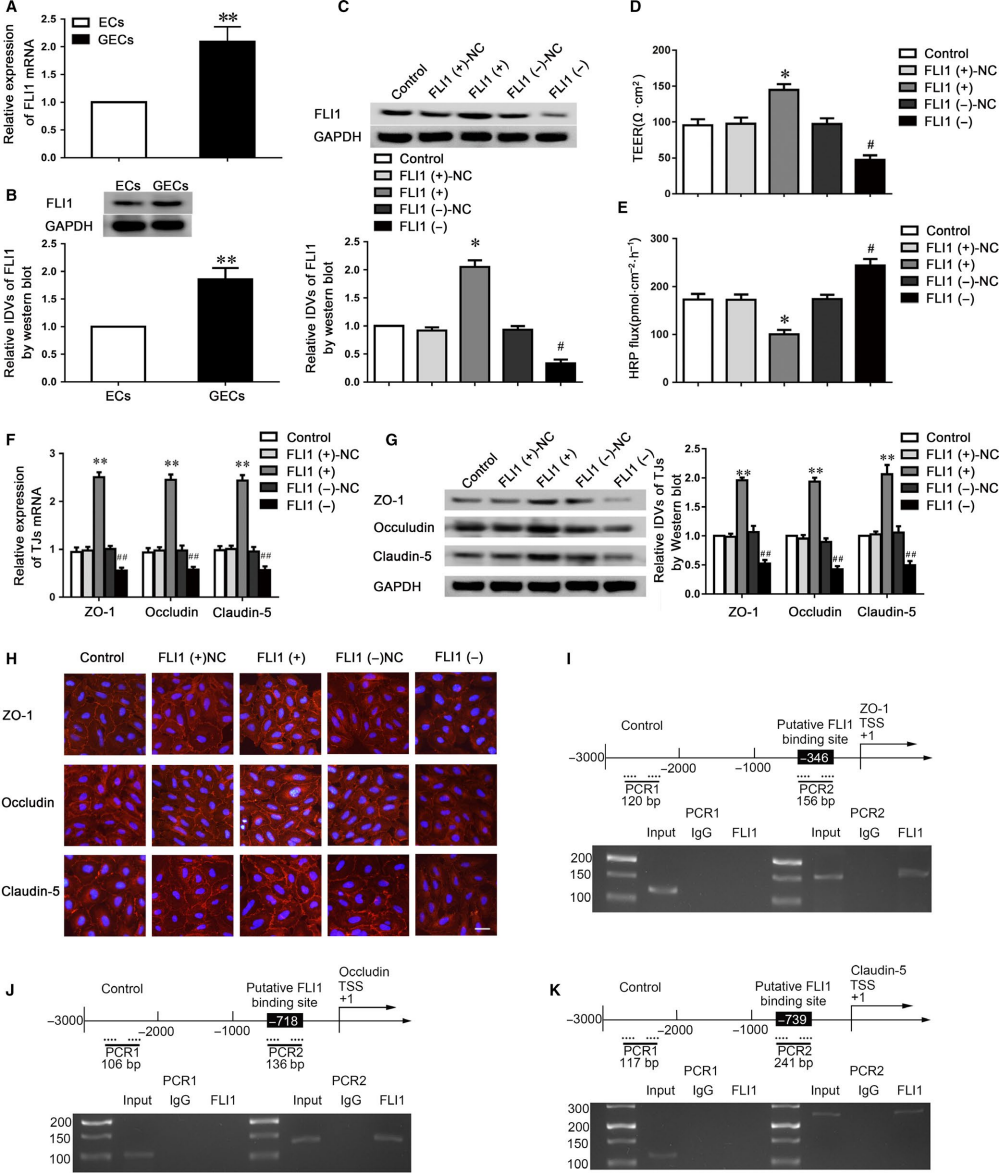
FLI1 modulated BTB permeability by regulating the expression of tight junction‐related proteins. Relative FLI1 expressions in ECs and GECs by qPCR (A) and Western blot assays (B). Data represented as mean ± SD (n = 5). **P < .01 vs. ECs group. C, FLI1 protein expressions were evaluated using Western blot assay in the GECs with FLI1 overexpression and knockdown. The BTB permeability and integrity was evaluated using TEER values (D) and HRP flux (E) in the BTB model with FLI1 overexpression and knockdown. The expression changes of ZO‐1, occludin, and claudin‐5 were determined using qRT‐PCR (F) and Western blot assays (G) in FLI1 overexpressed and knockdown GECs. Data represented as mean ± SD (n = 5). *P < .05, **P < .01 vs. FLI1 (+)‐NC. #*P* < .05, ##*P* < .01 vs. FLI1 (−)‐NC group. H, Immunofluorescent staining of tight junction‐related proteins in GECs in FLI1 overexpressed and knockdown GECs. Scale bar represents 20 μm. Schematic representation of the putative FLI1 binding sites in human ZO‐1 (I), occludin (J) and claudin‐5 (K) promoter regions and their respective ChIP PCR products were shown

MiR‐194‐5p may act as a post‐transcriptional regulator to regulate the BTB permeability via binding to the 3′UTR of target genes to repress protein production. By searching the TargetScan and miRanda bioinformatics database, FLI1 was predicted as a direct target of miR‐194‐5p. The FLI1 expression was first assessed in ECs and GECs. As indicated in Figure 4A,B, FLI1 expression was markedly increased in GECs compared with the ECs. We also overexpressed or knocked down FLI1 in GECs (Figure 4C) to determine the effects of FLI1 on BTB permeability. Compared to the FLI1(+) NC group, the FLI1(+) group performed the increased TEER value and reduced HRP flux. Opposite results were shown between the FLI1 (−) group and FLI1 (−)‐NC group (Figure 4D,E).

To further uncover the underlying mechanisms of FLI1 on the regulation of barrier permeability, we used qRT‐PCR and western blotting to investigate the mRNA and protein expressions of tight junction‐related proteins (Figure 4F,G). The mRNA and protein expressions of claudin‐5, occludin and ZO‐1 were markedly up‐regulated in the FLI1 (+) group and obviously down‐regulated in the FLI1 (−) group. Moreover, immunofluorescence analysis revealed that the discontinuous distributions of tight junction‐related proteins on the boundaries of cells were observed in the FLI1 (−) group (Figure 4H). To clarify whether FLI1 could directly bind to the promoters of claudin‐5, occludin and ZO‐1 in GECs, ChIP assays were conducted. FLI1 has been reported to bind to DNA through a consensus sequence GGAA/T.^22^ According to the DBTSS HOME database, we confirmed the promoter regions of claudin‐5, occludin and ZO‐1. By scanning the DNA sequence in the upstream 1500 bp and downstream 200 bp of transcription starting site (TSS), predicted FLI1 binding sites at −346 positions in ZO‐1, −718 positions in occludin and −739 positions in claudin‐5 were verified, respectively. Primers were designed to bind to the above FLI1 binding sequences as well as the negative control sequences in the upstream of the putative FLI1 binding sites, which was not predicted to bind to FLI1 (Table S3). The results demonstrated that FLI1 could bind to the putative binding sites of claudin‐5, occludin and ZO‐1, but not to the negative control groups (Figure 4I‐K).

### 3.5 FLI1 was involved in circ‐USP1‐ and miR‐194‐5p‐mediated regulation of BTB permeability as a target of miR‐194‐5p

**Figure 5:**
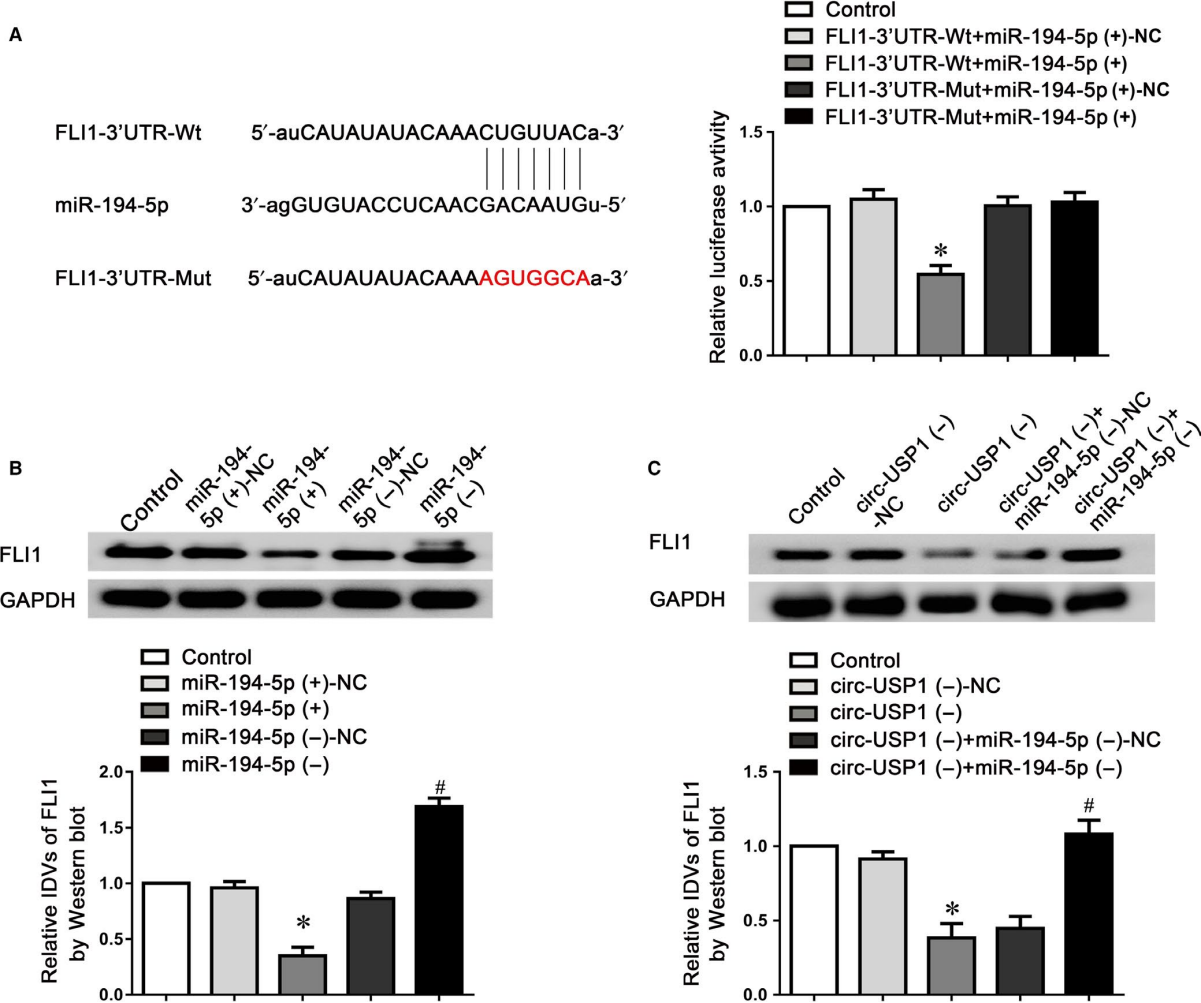
FLI1 was involved in circ‐USP1‐ and miR‐194‐5p‐mediated regulation of BTB permeability as a target of miR‐194‐5p. A, Relative luciferase activity was performed by dual‐luciferase reporter assay. Data represented as means ± SD (n = 5). *P < .05 vs. FLI1‐3′UTR‐Wt+miR‐194‐5p(+)‐NC group. B, Effects of miR‐194‐5p overexpression or knockdown on the expression of FLI1 by Western blot assay. Data represented as mean ± SD (n = 5). *P < .05 vs. miR‐194‐5p (+)‐NC group. #*P* < .05 vs. miR‐194‐5p (−)‐NC group. C, Western blot assay to evaluate the effect of circ‐USP1 and miR‐194‐5p knockdown on the expression of FLI1. Data represented as mean ± SD (n = 5). *P < .05 vs. circ‐USP1 (−)‐NC group. #*P* < .05 vs. circ‐USP1 (−)+miR‐194‐5p (−)‐NC group

To identify that FLI1 is a functional target of miR‐194‐5p, the dual‐luciferase reporter assay was conducted. Figure 5A indicated that the FLI1‐3′UTR‐Wt+miR‐194‐5p (+) group performed obviously weaker luciferase activity compared with the FLI1‐3′UTR‐Wt+miR‐194‐5p (+)‐NC group, whilst the luciferase activity in the FLI1‐3′UTR‐Mut+miR‐194‐5p (+) group remained unchanged. Furthermore, as indicated in Figure 5B, miR‐194‐5p overexpression markedly reduced FLI1 expressing level, whilst miR‐194‐5p knockdown showed the opposite effect. Moreover, FLI1 expression was markedly reduced in the circ‐USP1 (−) group, compared to the circ‐USP1 (−)‐NC group. However, knockdown of miR‐194‐5p significantly reversed the down‐regulated FLI1 protein in circ‐USP1 silenced GECs and almost restored the expression of FLI1 to a similar level as the control group (Figure 5C).

**Figure 6:**
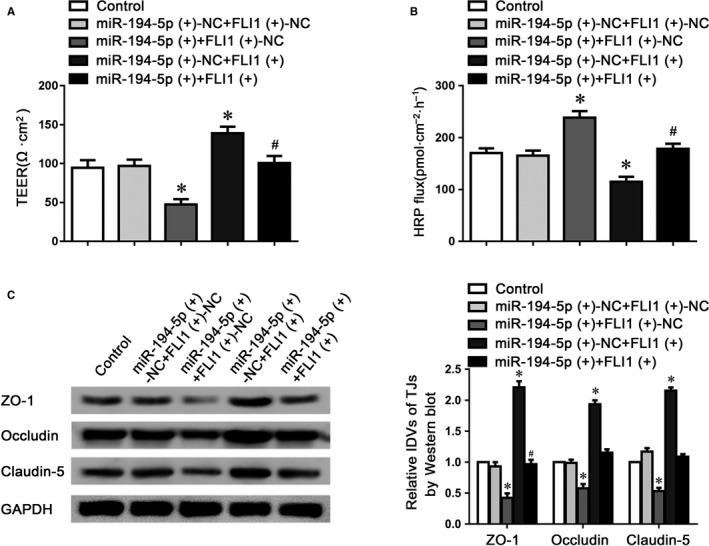
MiR‐194‐5p regulated BTB permeability via targeting FLI1. Effects of miR‐194‐5p and FLI1 on TEER values (A) and HRP flux (B) in the BTB model in vitro. C, Western bolt assay to evaluate the expression of tight junction‐related proteins with miR‐194‐5p overexpressed alone or combined with FLI1. Data represented as mean ± SD (n = 5). *P < .05 vs. miR‐194‐5p (+)‐NC+FLI1 (+)‐NC group. #*P* < .05 vs. miR‐194‐5p (+)+FLI1(+)‐NC group

To further clarify that miR‐194‐5p might regulate BTB permeability via targeting FLI1, TEER and HRP flux assays were performed respectively. As indicated in Figure 6A,B, the co‐overexpression of miR‐194‐5p and FLI1 significantly reversed miR‐194‐5p‐induced down‐regulation of TEER value, and up‐regulation of HRP flux, compared to the miR‐194‐5p (+) +FLI1 (+)‐NC group. Moreover, claudin‐5, occludin and ZO‐1 protein expressions were assessed using western blotting, which showed that the co‐overexpression of miR‐194‐5p and FLI1 rescued the down‐regulation of claudin‐5, occludin and ZO‐1 proteins via overexpressing miR‐194‐5p alone (Figure 6C). These data indicated that FLI1 could regulate miR‐194‐5p‐induced permeability changes of BTB.

### 3.6 Combined treatment of circ‐USP1 and miR‐194‐5p promoted doxorubicin delivery across BTB and induced apoptosis of glioma cells

**Figure 7:**
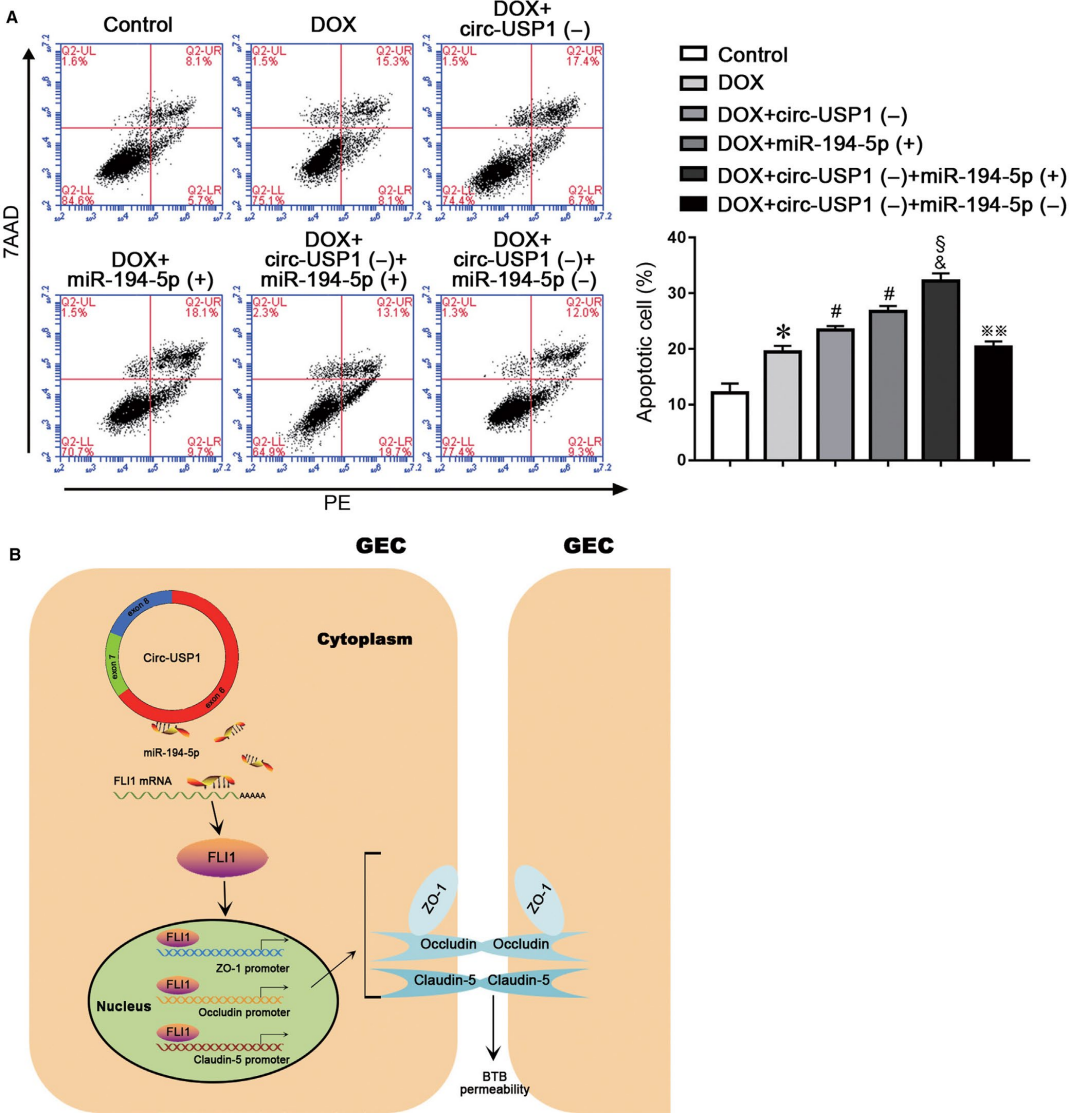
The apoptosis changes of U87 cells induced by combined treatment of circ‐USP1 and miR‐194‐5p with doxorubicin. A, Flow cytometry analysis detected the apoptosis rates of U87 cells in different groups. Data represented as mean ± SD (n = 3). *P < .05 vs. control group, #*P* < .05 vs. DOX group, §*P* < .05 vs. DOX+circ‐USP1(−) group, &*P* < .05 vs. DOX+miR‐194‐5p(+) group, ※※*P* < .01 vs. DOX+circ‐USP1(−)+miR‐194‐5p(+) group. B, The schematic diagram of the mechanism by which circ‐USP1/miR‐194‐5p axis regulates the BTB permeability

In order to clarify whether the treatment of circ‐USP1 and miR‐194‐5p alone or in combination could increase the BTB permeability and promote the anti‐tumour drug doxorubicin (DOX) delivery across BTB to induce the apoptosis of U87 glioma cells. The apoptosis rates of U87 cells were detected with different treatment. The apoptosis rates in the DOX+circ‐USP1(−) group and the DOX+miR‐194‐5p (+) group were significantly higher than the DOX group. Moreover, compared with the DOX+circ‐USP1(−) group or the DOX+miR‐194‐5p (+) group, the apoptosis rate of U87 cells increased significantly in the DOX+circ‐USP1(−)+miR‐194‐5p(+) group. However, the DOX+circ‐USP1(−)+miR‐194‐5p(−) treatment could significantly reverse the up‐regulated apoptosis rate of U87 cells induced by the DOX+circ‐USP1(−)+miR‐194‐5p(+) treatment. The above results indicate that the treatment of circ‐USP1 and miR‐194‐5p alone or in combination could effectively promote DOX delivery across BTB and induce the apoptosis of glioma cells. Taken together, circ‐USP1 knockdown combined with miR‐194‐5p overexpression showed better DOX delivery effects across BTB than the treatment of circ‐USP1 and miR‐194‐5p alone (Figure 7A). A schematic representation of the mechanism by which the circ‐USP1/miR‐194‐5p axis regulates the BTB permeability is presented in Figure 7B.

## 4 DISCUSSION

In our study, circ‐USP1 was highly enriched in GECs. Knockdown of circ‐USP1 down‐regulated claudin‐5, occludin and ZO‐1 protein expressions, thus increased BBB permeability, impaired its integrity. On the contrary, miR‐194‐5p expression was reduced in GECs. Overexpression of miR‐194‐5p up‐regulated the BTB permeability and reduced the expressions of tight junction‐related proteins. Circ‐USP1 bound to miR‐194‐5p. FLI1 was highly enriched in GECs and was a direct and functional target of miR‐194‐5p. Knockdown of FLI1 down‐regulated claudin‐5, occludin and ZO‐1 in both mRNA and protein levels, increased BTB permeability. FLI1 bound directly to the promoters of claudin‐5, occludin and ZO‐1 and acted as a transcriptional regulator of these genes. FLI1 was involved in circ‐USP1/miR‐194‐5p‐mediated barrier permeability regulation. The results above clarified for the first time that circ‐USP1 modulated BTB permeability via a miR‐194‐5p/FLI1‐mediated pathway.

Recently, increasing evidence proposes that circRNAs play a crucial role in the initiation and development of cancer.^32^, ^33^ Studies have shown that circRNA ZNF292 silencing blocked glioma cell cycle progression through the regulation of Wnt/beta‐catenin signalling pathway, thus suppressed gliomas cells proliferation.^34^ CircRNAs are also abundantly enriched in vascular endothelial cells, however, the function of circRNAs remains unknown. In endothelial cells, cZNF292 was identified as the highest expressed circRNAs. Depletion of cZNF292 inhibited angiogenic sprouting of endothelial cells suggested that cZNF292 showed a pro‐angiogenic function.^35^ In our study, circ‐USP1 was highly expressed in GECs, whilst no significant difference was observed in the expression of lin‐USP1 between ECs and GECs. Researchers verified that the expression of some circular isoforms, such as the circRNAs derived from the DCC (deleted in colorectal cancer) gene, diversified across a range of human tissues and was not correlated with its cognate linear mRNA expression.^36^ Because of the up‐regulation of circ‐USP1 in GECs, we are interested to explore whether the dysregulation of circ‐USP1 was associated with the regulation of BTB function. The down‐regulation of tight junction‐related proteins ZO‐1, occludin and claudin‐5 is a landmark change of BTB permeability via the paracellular pathway.^30^, ^31^, ^37^ Knockdown of circ‐USP1 reduced the expression of claudin‐5, occludin and ZO‐1, thus up‐regulated BTB permeability. These data indicated that circ‐USP1 is involved in the regulation of BTB permeability.

We further explored the possible mechanisms of circ‐USP1 in the regulation of barrier function. CircRNAs may act as a miRNA sponge to bind to miRNAs, and miRNAs would tether RISC to the circRNAs. The interaction between miRNA and circRNA may competitively affect miRNA‐mediated regulation of their target genes.^38^, ^39^, ^40^ In oral squamous cell carcinoma cells, circ_100290 plays oncogene function by binding with miR‐29b.^41^ We demonstrated that circ‐USP1 may interact with miR‐194‐5p and function as an endogenous miR‐194‐5p sponge. Knockdown of circ‐USP1 may result in the release of miR‐194‐5p and improved its activity. MiR‐194 is a kind of the p53 responsive miRNAs, which has been reported to significantly suppress the proliferation and invasion of several cancer cells and acts as a tumour suppressor.^42^, ^43^ MiR‐194‐5p has been found to be low expressed in glioma tissue and overexpression of miR‐194‐5p suppresses invasion and epithelial‐mesenchymal transition of glioma cells.^44^ Under serum‐deprived condition, miR‐194‐5p was involved in the regulation of endothelial gene expression as well as the functional angiogenic activity.^21^ In GECs, we found that miR‐194‐5p was low expressed. Increased miR‐194‐5p reduced claudin‐5, occludin and ZO‐1 expressing levels, and up‐regulated BTB permeability, which indicated that miR‐194‐5p may function as a novel regulator of BTB permeability.

In addition, bioinformatics analysis and luciferase reporter assay demonstrated that FLI1 acted as a target of miR‐194‐5p. Overexpression of FLI1 reversed the miR‐194‐5p‐induced increase of BTB permeability, which suggested FLI1 played a crucial role in miR‐194‐5p‐mediated BBB permeability regulation. As a member of the ETS transcription factor family, FLI1 was known to regulate the expression of oncogenes, tumour suppressor genes, and some genes involved in maintaining vascular homoeostasis such as VE‐cadherin, platelet endothelial cell adhesion molecule 1.^26^, ^28^, ^45^ In GECs, we found that FLI1 was highly expressed and knockdown of FLI1 down‐regulated claudin‐5, occludin and ZO‐1 expression, which increased BTB permeability. Similarly, knockdown of FLI1 and ETS‐related gene, its closest homolog, increased human pulmonary endothelial cell monolayer permeability with capacity like that of vascular endothelial growth factor. Meanwhile, the genes involved in the regulation endothelial homoeostasis and cell‐cell adhesion were reduced.^28^ Another finding inconsistent with our results is that in mice with a conditional deletion of FLI1 in endothelial cells exhibited disorganized dermal vasculature with obviously compromised vessel integrity and significantly increased vessel permeability.^45^ In this study, overexpression of FLI1 up‐regulated claudin‐5, occludin and ZO‐1 in both mRNA and protein levels. Using CHIP assays, FLI1 was demonstrated to bind directly to the promoters of tight junction‐associated proteins claudin‐5, occludin and ZO‐1, indicating that FLI1 promoted the transcription of genes related to the regulation of BTB permeability.

The anthracyclines anti‐tumour antibiotic doxorubicin (Dox) is widely used in the chemotherapy of various types of cancers. For the treatment of brain tumours, the existence of the blood‐tumour barrier restricts Dox to enter the brain and reach a therapeutic concentration. To further evaluate the regulatory function of BTB permeability by treatment of circ‐USP1 and miR‐194‐5p alone or in combination, Dox was treated with the above regulatory factors. It was demonstrated that circ‐USP1 knockdown combined with miR‐194‐5p overexpression significantly increased the apoptosis rate of U87 glioma cells induced by Dox, compared with the circ‐USP1 knockdown or miR‐194‐5p overexpression alone, suggesting that the treatment of circ‐USP1 and miR‐194‐5p in combination could enhance Dox‐induced anti‐tumour effect by promoting its penetrating capability across BTB.

Taken together, this study for the first time showed that highly expressed circ‐USP1 acts as a regulator of BTB permeability. Knockdown of circ‐USP1 impaired the BTB integrity increased BTB permeability via binding to miR‐194‐5p. The overexpressed miR‐194‐5p targeted transcription factor FLI1 to negatively regulate its expression, which resulted in the down‐regulation of claudin‐5, occludin and ZO‐1. In summary, the circ‐USP1/miR‐194‐5p/FLI1 pathway plays a crucial role in regulating BTB functions.

## CONFLICT OF INTEREST

All authors declare no conflicts of interest.

## AUTHOR CONTRIBUTIONS

Ping Wang, Yixue Xue and Yunhui Liu designed the study. Yang Gao, Peiqi Wu, Yawen Ma, Xiaobai Liu and Qianru He performed the experiments and acquired the data. Yang Gao, Peiqi Wu, Yawen Ma, Jian Zheng, Xiaobai Liu, Jun Ma and Libo Liu analysed and interpreted the acquired data. Ping Wang, Yang Gao, Peiqi Wu and Yixue Xue participated in discussion and writing of the manuscript. All authors read and approved the final manuscript.

## Data Availability

The data that support the findings of this study are available from the corresponding author upon reasonable request.

## References

[1] Jain KK . A critical overview of targeted therapies for glioblastoma. Front Oncol. 2018;8:419. 30374421 10.3389/fonc.2018.00419 PMC6196260

[2] Dubois LG , Campanati L , Righy C , et al. Gliomas and the vascular fragility of the blood brain barrier. Front Cell Neurosci. 2014;8:418. 25565956 10.3389/fncel.2014.00418 PMC4264502

[3] Wang Y , Liu J , Ma J , et al. Exosomal circRNAs: biogenesis, effect and application in human diseases. Mol Cancer. 2019;18(1):116. 31277663 10.1186/s12943-019-1041-z PMC6610963

[4] Su M , Xiao Y , Ma J , et al. Circular RNAs in cancer: emerging functions in hallmarks, stemness, resistance and roles as potential biomarkers. Mol Cancer. 2019;18(1):90. 30999909 10.1186/s12943-019-1002-6 PMC6471953

[5] Hansen TB , Jensen TI , Clausen BH , et al. Natural RNA circles function as efficient microRNA sponges. Nature. 2013;495(7441):384‐388. 23446346 10.1038/nature11993

[6] Ding S , Zhu Y , Liang Y , Huang H , Xu Y , Zhong C . Circular RNAs in vascular functions and diseases. Adv Exp Med Biol. 2018;1087:287‐297. 30259375 10.1007/978-981-13-1426-1_23

[7] Bayoumi AS , Aonuma T , Teoh JP , Tang YL , Kim IM . Circular noncoding RNAs as potential therapies and circulating biomarkers for cardiovascular diseases. Acta Pharmacol Sin. 2018;39(7):1100‐1109. 29565037 10.1038/aps.2017.196 PMC6289320

[8] He Q , Zhao L , Liu X , et al. MOV10 binding circ‐DICER1 regulates the angiogenesis of glioma via miR‐103a‐3p/miR‐382‐5p mediated ZIC4 expression change. J Exp Clin Cancer Res. 2019;38(1):9. 30621721 10.1186/s13046-018-0990-1 PMC6323715

[9] Liu C , Yao MD , Li CP , et al. Silencing of circular RNA‐ZNF609 ameliorates vascular endothelial dysfunction. Theranostics. 2017;7(11):2863‐2877. 28824721 10.7150/thno.19353 PMC5562221

[10] Lee JK , Chang N , Yoon Y , et al. USP1 targeting impedes GBM growth by inhibiting stem cell maintenance and radioresistance. Neuro‐Oncology. 2016;18(1):37‐47. 26032834 10.1093/neuonc/nov091 PMC4677407

[11] Kulkarni B , Kirave P , Gondaliya P , et al. Exosomal miRNA in chemoresistance, immune evasion, metastasis and progression of cancer. Drug Discov Today. 2019;6446(19):30033‐30039. 10.1016/j.drudis.2019.06.010 31228614

[12] Piperigkou Z , Karamanos NK . Dynamic interplay between miRNAs and the extracellular matrix influences the tumor microenvironment. Trends Biochem Sci. 2019;0004(19):30140‐30149. 10.1016/j.tibs.2019.06.007 31288968

[13] Miao YS , Zhao YY , Zhao LN , et al. MiR‐18a increased the permeability of BTB via RUNX1 mediated down‐regulation of ZO‐1, occludin and claudin‐5. Cell Signal. 2015;27(1):156‐167. 25452107 10.1016/j.cellsig.2014.10.008

[14] Ma J , Wang P , Yao Y , et al. Knockdown of long non‐coding RNA MALAT1 increases the blood‐tumor barrier permeability by up‐regulating miR‐140. Biochim Biophys Acta. 2016;1859(2):324‐338. 26619802 10.1016/j.bbagrm.2015.11.008

[15] Sa L , Li Y , Zhao L , et al. The role of HOTAIR/miR‐148b‐3p/USF1 on regulating the permeability of BTB. Front Mol Neurosci. 2017;10:194. 28701916 10.3389/fnmol.2017.00194 PMC5487514

[16] Liu L , Shi Y , Shi J , et al. The long non‐coding RNA SNHG1 promotes glioma progression by competitively binding to miR‐194 to regulate PHLDA1 expression. Cell Death Dis. 2019;10(6):463. 31189920 10.1038/s41419-019-1698-7 PMC6561933

[17] Tang H , Zhao H , Yu ZY , et al. MicroRNA‐194 inhibits cell invasion and migration in hepatocellular carcinoma through PRC1‐mediated inhibition of Wnt/β‐catenin signaling pathway. Dig Liver Dis. 2019;51(9):1314‐1322. 30948333 10.1016/j.dld.2019.02.012

[18] Wang SH , Wu XC , Zhang MD , et al. Long noncoding RNA H19 contributes to gallbladder cancer cell proliferation by modulated miR‐194‐5p targeting AKT2. Tumour Biol. 2016;37(7):9721‐9730. 26803515 10.1007/s13277-016-4852-1

[19] Dell'Aversana C , Giorgio C , D'Amato L , et al. miR‐194‐5p/BCLAF1 deregulation in AML tumorigenesis. Leukemia. 2017;31(11):2315‐2325. 28216661 10.1038/leu.2017.64 PMC5668498

[20] Zhu X , Li D , Yu F , et al. miR‐194 inhibits the proliferation, invasion, migration, and enhances the chemosensitivity of non‐small cell lung cancer cells by targeting forkhead box A1 protein. Oncotarget. 2016;7(11):13139‐13152. 26909612 10.18632/oncotarget.7545 PMC4914347

[21] Brumm AJ , Nunez S , Doroudchi MM , et al. Astrocytes can adopt endothelial cell fates in a p53‐dependent manner. Mol Neurobiol. 2017;54(6):4584‐4596. 27389775 10.1007/s12035-016-9974-3 PMC5219956

[22] Lennard RM , Nowling TK , Brandon D , Watson DK , Zhang XK . Fli‐1 controls transcription from the MCP‐1 gene promoter, which may provide a novel mechanism for chemokine and cytokine activation. Mol Immuno. 2015;63(2):566‐573. 10.1016/j.molimm.2014.07.013 PMC4254164 25108845

[23] Ben‐David Y , Giddens EB , Bernstein A . Identification and mapping of a common proviral integration site Fli‐1 in erythroleukemia cells induced by Friend murine leukemia virus. Proc Natl Acad Sci USA. 1990;87(4):1332‐1336. 2304901 10.1073/pnas.87.4.1332 PMC53469

[24] Osgood CL , Tantawy MN , Maloney N , et al. 18F‐FLT Positron Emission Tomography (PET) is a pharmacodynamic marker for EWS‐FLI1 activity and Ewing sarcoma. Sci Rep. 2016;6:33926. 27671553 10.1038/srep33926 PMC5037393

[25] Song W , Li W , Li L , et al. Friend leukemia virus integration 1 activates the Rho GTPase pathway and is associated with metastasis in breast cancer. Oncotarget. 2015;6(27):23764‐23775. 26156017 10.18632/oncotarget.4350 PMC4695150

[26] Tsai HP , Tsai TH , Hsieh YJ , et al. Overexpression of Fli‐1 in astrocytoma is associated with poor prognosis. Oncotarget. 2017;8(17):29174‐29186. 28418872 10.18632/oncotarget.16303 PMC5438722

[27] Abedin MJ , Nguyen A , Jiang N , et al. Fli1 acts downstream of Etv2 to govern cell survival and vascular homeostasis via positive autoregulation. Circ Res. 2014;114(11):1690‐1699. 24727028 10.1161/CIRCRESAHA.1134303145 PMC4080722

[28] Looney AP , Han R , Stawski L , Marden G , Iwamoto M , Trojanowska M . Synergistic role of endothelial ERG and FLI1 in mediating pulmonary vascular homeostasis. Am J Respir Cell Mol Biol. 2017;57(1):121‐131. 28248553 10.1165/rcmb.2016-0200OC PMC5516275

[29] Ma J , Wang P , Liu Y , Zhao L , Li Z , Xue Y . Kruppel‐like factor 4 regulates blood‐tumor barrier permeability via ZO‐1, occludin and claudin‐5. J Cell Physiol. 2014;229(7):916‐926. 24318462 10.1002/jcp.24523

[30] Cai H , Liu W , Xue Y , et al. Roundabout 4 regulates blood‐tumor barrier permeability through the modulation of ZO‐1, occludin, and claudin‐5 expression. J Neuropathol Exp Neurol. 2015;74(1):25‐37. 25470344 10.1097/NEN.0000000000000146

[31] Zhao L , Wang P , Liu Y , Ma J , Xue Y . miR‐34c regulates the permeability of blood‐tumor barrier via MAZ‐mediated expression changes of ZO‐1, occludin, and claudin‐5. J Cell Physiol. 2015;230(3):716‐731. 25201524 10.1002/jcp.24799

[32] Shen B , Wang Z , Li Z , Song H , Ding X . Circular RNAs: an emerging landscape in tumor metastasis. Am J Cancer Res. 2019;9(4):630‐643. 31105992 PMC6511637

[33] Haddad G , Lorenzen JM . Biogenesis and function of circular RNAs in health and in disease. Front Pharmacol. 2019;10:428. 31080413 10.3389/fphar.2019.00428 PMC6497739

[34] Yang P , Qiu Z , Jiang Y , et al. Silencing of cZNF292 circular RNA suppresses human glioma tube formation via the Wnt/beta‐catenin signaling pathway. Oncotarget. 2016;7(39):63449‐63455. 27613831 10.18632/oncotarget.11523 PMC5325376

[35] Boeckel JN , Jaé N , Heumüller AW , et al. Identification and characterization of hypoxia‐regulated endothelial circular RNA. Circ Res. 2015;117(10):884‐890. 26377962 10.1161/CIRCRESAHA.115.306319

[36] Nigro JM , Cho KR , Fearon ER , et al. Scrambled exons. Cell. 1991;64(3):607‐613. 1991322 10.1016/0092-8674(91)90244-s

[37] Guo J , Cai H , Zheng J , et al. Long non‐coding RNA NEAT1 regulates permeability of the blood‐tumor barrier via miR‐181d‐5p‐mediated expression changes in ZO‐1, occludin, and claudin‐5. Biochim Biophys Acta Mol Basis Dis. 2017;1863(9):2240‐2254. 28185956 10.1016/j.bbadis.2017.02.005

[38] Lu Q , Liu T , Feng H , et al. Circular RNA circSLC8A1 acts as a sponge of miR‐130b/miR‐494 in suppressing bladder cancer progression via regulating PTEN. Mol Cancer. 2019;18(1):111. 31228937 10.1186/s12943-019-1040-0 PMC6588875

[39] Zheng SQ , Qi Y , Wu J , et al. CircPCMTD1 acts as the sponge of miR‐224‐5p to promote glioma progression. Front Oncol. 2019;9:398. 31179240 10.3389/fonc.2019.00398 PMC6538694

[40] Zheng Q , Bao C , Guo W , et al. Circular RNA profiling reveals an abundant circHIPK3 that regulates cell growth by sponging multiple miRNAs. Nat Commun. 2016;7:11215. 27050392 10.1038/ncomms11215 PMC4823868

[41] Chen L , Zhang S , Wu J , et al. circRNA_100290 plays a role in oral cancer by functioning as a sponge of the miR‐29 family. Oncogene. 2017;36(32):4551‐4561. 28368401 10.1038/onc.2017.89 PMC5558096

[42] Sundaram P , Hultine S , Smith LM , et al. p53‐responsive miR‐194 inhibits thrombospondin‐1 and promotes angiogenesis in colon cancers. Cancer Res. 2011;71(24):7490‐7501. 22028325 10.1158/0008-5472.CAN-11-1124 PMC3242824

[43] Pichiorri F , Suh SS , Rocci A , et al. Downregulation of p53‐inducible microRNAs 192, 194, and 215 impairs the p53/MDM2 autoregulatory loop in multiple myeloma development. Cancer Cell. 2010;18(4):367‐381. 20951946 10.1016/j.ccr.2010.09.005 PMC3561766

[44] Zhang X , Wei C , Li J , Liu J , Qu J . MicroRNA‐194 represses glioma cell epithelial‐to‐mesenchymal transition by targeting Bmi1. Oncol Rep. 2017;37:1593‐1600. 28098896 10.3892/or.2017.5376

[45] Asano Y , Stawski L , Hant F , et al. Endothelial Fli1 deficiency impairs vascular homeostasis: a role in scleroderma vasculopathy. Am J Pathol. 2011;176(4):1983‐1998. 10.2353/ajpath.2010.090593 PMC2843486 20228226

