# Supplementary material for: Circular RNA USP1 regulates the permeability of blood‐tumour barrier via miR‐194‐5p/FLI1 axis
Source: J Cell Mol Med. 2019 Oct 26;24(1):342–55. doi: 10.1111/jcmm.14735 (PMC6933377; doi:10.1111/jcmm.14735)
Supplement: Supplementary file 1 [file JCMM-24-342-s001.pdf]

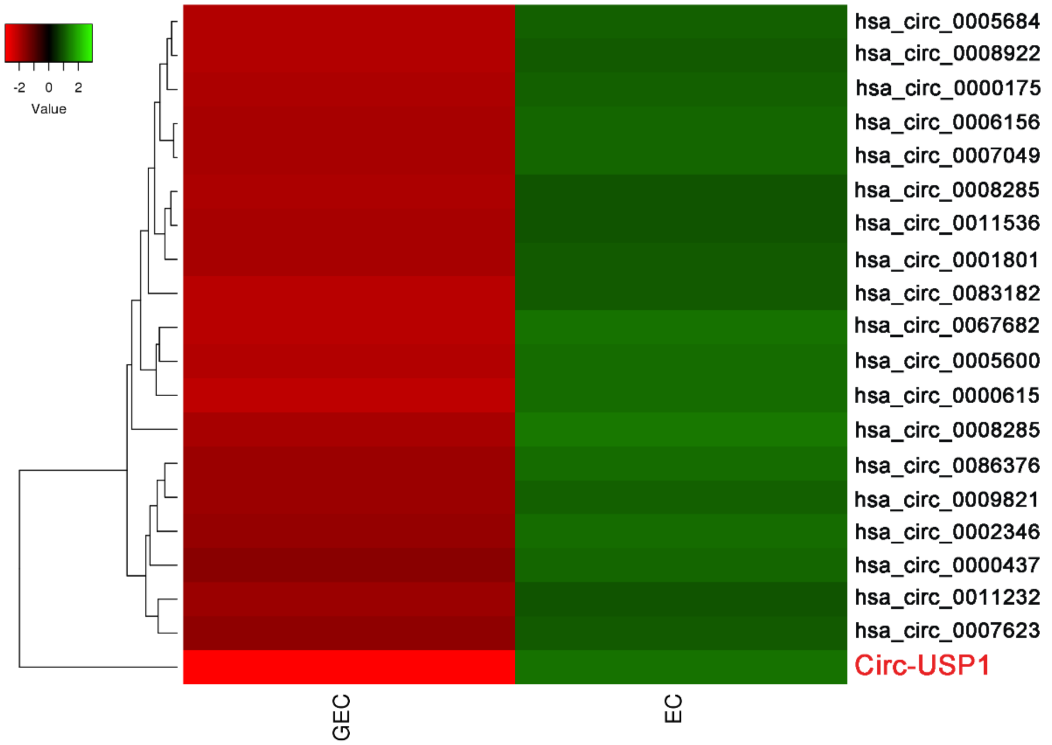

Figure S1. The heatmap of top 20 upregulated circular RNAs in GECs.

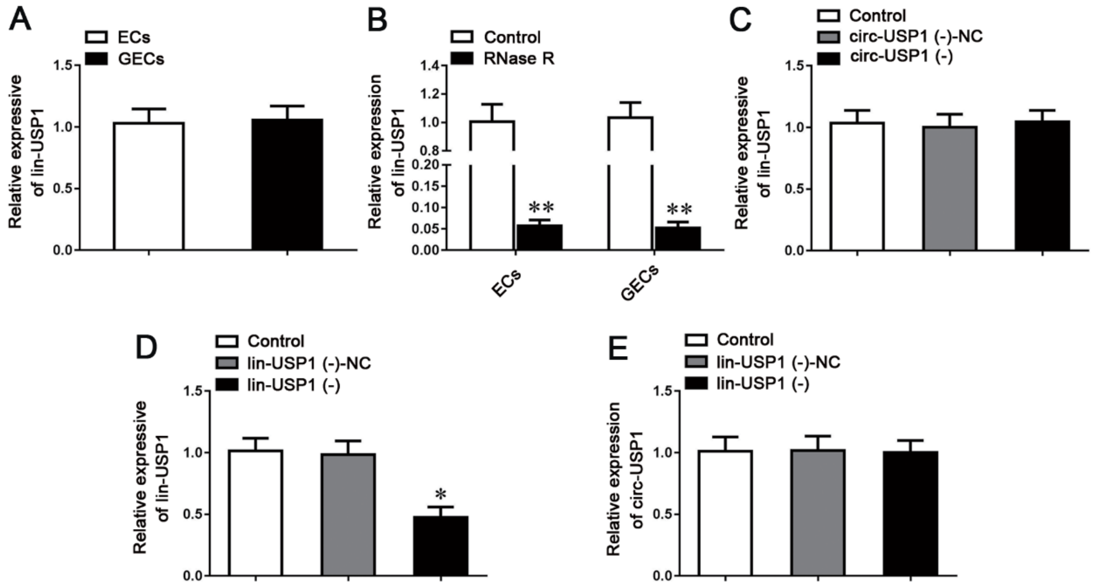

Figure S2. The expressions of lin-USP1 in ECs and GECs and the effects of circ-USP1 on lin-USP1 expression. (A) Relative expression of lin-UPS1 in ECs and GECs by qRT-PCR. (B) Relative expression of lin-UPS1 with RNase R treatment in ECs and GECs by qRT-PCR. Data represented as mean ± SD (n=5). ***P*<0.01 vs. control group. (C) Relative expression of lin-UPS1 with circ-USP1 knockdown in the GECs was evaluated using qRT-PCR. (D) Relative expression of lin-UPS1 with lin-UPS1 knockdown in the GECs was detected by qRT-PCR. Data represented as mean ± SD (n=5). **P*<0.05 vs. lin-UPS1 (-)-NC group. (E) Relative expression of circ-UPS1 with lin-UPS1 knockdown in the GECs was detected by qRT-PCR.
