# Supplementary material for: Circular RNA USP1 regulates the permeability of blood‐tumour barrier via miR‐194‐5p/FLI1 axis
Source: J Cell Mol Med. 2019 Oct 26;24(1):342–55. doi: 10.1111/jcmm.14735 (PMC6933377; doi:10.1111/jcmm.14735)
Supplement: Supplementary file 2 [file JCMM-24-342-s002.pdf]

Table S1. Primers and probes used for qRT-PCR.

Gene	Sequence (5'->3') or Assay ID
Circ-USP1	F: TAACTATTCATTTGAAGTGCTTTGC<br>R: GGCATGTTTCTTGAATGTTTCC<br>Probe:<br>FAM+CAGCGCTAGTGGTTTGGAGGAACT<br>CAACCCTATGCGCTG+DABCYI
GAPDH	F: GGACCTGACCTGCCGTCTAG<br>R: TAGCCCAGGATGCCCTTGAG<br>Probe:<br>FAM+CCTCCGACGCCTGCTTCACCACCT<br>+Eclipse
Lin-USP1	F: GCTCTGTGCCTGCGTTGTT<br>R: TCTCCACGCTGCAGAGACAC
GAPDH	F: GGACCTGACCTGCCGTCTAG<br>R: TAGCCCAGGATGCCCTTGAG
U6	001973 (Applied biosystems)
MiR-194-5p	000493 (Applied biosystems)

Table S2. Sequences of shRNA template

Gene		Sequence (5'->3')
Circ-USP1	Sence	CACCGCTAGTGGTTTGGAGGAACTCATTCAAGA<br>GATGAGTTCCTCCAAACCACTAGTTTTTG
	Antisence	GATCCAAAAAACTAGTGGTTTGGAGGAACTCAT<br>CTCTTGAATGAGTTCCTCCAAACCACTAGC
FLI1	Sence	CACCGGGCACAAACGATCAGTAAGATTCAAGA<br>GATCTTACTGATCGTTTGTGCCCTTTTTG
	Antisence	GATCCAAAAAGGGCACAAACGATCAGTAAGA<br>TCTCTTGAATCTTACTGATCGTTTGTGCCC
NC	Sence	CACCGTTCTCCGAACGTGTCACGTCAAGAGATT<br>ACGTGACACGTTCGGAGAATTTTTG
	Antisence	GATCCAAAAAATTCTCCGAACGTGTCACGTAAT<br>CTCTTGACGTGACACGTTCGGAGAAC

Table S3. Primers used for ChIP experiments

Gene	Binding site or Control	Sequence (5'->3')	Product size (bp)	Annealing temperature (°C)
ZO-1	PCR1	F: AAACGTGAGCAACTACGC	120	50.0
		R: TGAGTCCAGATCGTGCC		
	PCR2	F: AGAGCAACGCTTCTGACCCT	156	53.4
		R: ACCCGACAGTTGTTTCCTTCAC		
Occludin	PCR1	F: ACTGTGAGTTGCCAAGAAG	106	56.0
		R: GTTAAATGATGCCATGCTC		
	PCR2	F: AAGCGGGTGGGATTGGATAG	136	50.0
		R: GTGGCAATTATAGTTCTTGGC		
Claudin-5	PCR1	F: CCAGTGAGGAACTAAGGCAGAG	117	50.0
		R: TGCGGGCATTGTTGTTACTCTT		
	PCR2	F:GAGGAGATGTCCAGGAGGGTCTGT	241	56.0
		R:GGTAAATGAGATAAGGCCAGCGT		
